# American Dental Association

**Published:** 1894-11

**Authors:** 


### AMERICAN DENTAL ASSOCIATION.

### DENTAL EDUCATION.[1]

Dr. Louis Ottofy, chairman of the section, read the report, an abstract of which follows:

No section reports having been made to the Association in 1893, the present report covers a period of two years. During that time the following dental colleges have been established: Atlanta Dental College, Atlanta, Ga.; Birmingham Dental College, Birmingham, Ala.; Ohio Medical University, Department of Dentistry, Columbus, Ohio; University College of Medicine, Department of Dentistry, Richmond, Va.; Marion Sims College of Medicine, Dental Department, St. Louis, Mo.; Tacoma College of Dental Surgery, Tacoma, Wash., and Milwaukee Medical College, School of Dentistry, Mil-

---

[1] Portion of discussion of American Dental Association, Old Point Comfort, August, 1894. Copied from *Dental Cosmos*, October number.

waukee, Wis. The following have been discontinued: United States Dental College, and Tooth-Saving Dental College, both of Chicago, leaving forty-four schools in active operation and granting degrees.

The present dental-school system of the United States presents more favorable conditions than at any time in the past, for which the National Association of Dental Faculties is largely responsible, practically all the colleges, whether members of the organization or not, being governed by its rules. During the past ten years the number of dental schools has doubled, and the tendency, under the lax individual laws, each State being a sovereign, is to still increase. If every new college has as its motive the education of students in a more thorough and efficient manner, all encouragement should be given. At present the fad is to establish dental departments in connection with existing medical schools, which is usually an inexpensive way, requiring only the addition of three or four dentists to the Faculty and the setting aside of two rooms in the college building for operative and technical purposes. While it adds revenue to the medical college, it does not usually add to the lustre of the profession. It is not desired to deprecate this condition, for many of the dental schools operated in conjunction with medical colleges are among the best of the country; but there are two hundred and fifty medical colleges in the land, and the addition of a vermiform appendix to each might foster a dangerous disease.

A radical change in the requirements for the admission of students is required. The method in vogue—of allowing each school to judge of the fitness of candidates—is faulty. The section would urge that the degree of Bachelor of Arts or its equivalent, obtained from a reputable university, should be requisite. The next important step which should be taken is the extension of the school-year to nine months, and of the complete course to four school-years.

Referring to the laws recently enacted by various foreign governments excluding graduates from American dental schools, the report maintained that as far as ability to actually benefit those in need of dental services is concerned, the American graduate is not equalled by the product of any foreign dental school in the world. In view of the restrictions placed about American dentists endeavoring to practise in foreign lands, it is only proper that we should demand from foreigners coming as practitioners or students compliance with the rules and customs here, especially a thorough acquaintance with the language; teaching and examinations should be conducted in English only, and credentials presented by foreigners should be closely scrutinized.

The two years have been prolific in papers on dental education. It has been suggested that a dental college cannot do justice to a larger number of students than teachers and demonstrators can come into actual contact with, because the men would not have sufficient actual practice. It would seem, in some instances, that the number of teachers, or at least of the demonstrators, should be increased, while where it is impossible to supply sufficient clinical material the student should be advised to obtain privileges for practical work under the tutelage of practitioners.

As to whether it is advisable for a student to change from one school to another, the disadvantages of changing outweigh the advantages, and the better course would be for the student to select a good school and begin and end his course there.

The habit of undergraduates engaging in practice is an evil which ought to be readily controlled by requiring every student to sign, before being permitted to matriculate, a contract, which legal advice says can be enforced in most of the States, embodying a statement that he has not been for a period of two years engaged or employed or interested in a practice conducted on principles not in accord with the accepted code, and has not violated any dental law during that period; and an agreement to conduct himself according to the code, to obey the dental law, and not engage in practice unlawfully; any violation of the agreement shall entitle the college to erase his name from its records and report its action to the other colleges; that his diploma shall be held during good behavior only, the college having the right to cancel it and remove his name from the list of graduates for conduct not in accordance with true professional ethics.

Such a requirement would attract a better element, and, coupled with the exaction of a high preliminary education referred to, would exert a wholesome influence upon the entire dental fabric of America.

The section could see no advantage in a change of the degree which has been mooted. One of the most important steps in dental education is about to be taken, in the organization of an association by the technical teachers. Dental technics, introduced a few years ago by Dr. G. V. Black, will be an absolute necessity of the dental student's education.

The report referred approvingly to the use of outlines or questions and answers to subjects insufficiently covered by the textbooks; to post-graduate teaching as carried on by some of the colleges and by the Post-Graduate Association. The Universities of Michigan and Minnesota have established a regular course covering a year for graduates who desire to further improve themselves in any particular branch.

The section regretted that opportunity was not found at the World's Columbian Dental College to take steps looking towards the adoption of a uniform nomenclature.

Adverting to the means of educating the public in dental matters, the report commended a pamphlet gotten out under the auspices of the Isaac Knapp Dental Coterie, of Fort Wayne, Indiana, but it believes the best way to impart this information is by parcelling it out in small doses and at frequent intervals, giving a single subject at a time. As a model of the kind of reports the section would be glad to receive from all the dental societies, it presented the report sent in by the Washington City Dental Society, giving an account of its work during the year. A list of the dental books published was presented, with brief comments on some of the more important works, and the report closed with an announcement of the other papers to be presented by the section.

*Dr. Frank Abbott.*—The disposition in all educational institutions connected with dentistry at present is not only to make their teaching more thorough, but to extend the time of work. They are now considering the advisability of extending the courses to four years. In New York State it has been considered better in professional education to begin at the other end; better to prepare a man to receive the professional teaching before he commences to pursue the special studies. The Regents of the University of New York deem this a better plan to secure a high standard than the extending of the qualifications required at the other end of the term of study. We can train a man pretty well in dentistry under the rules now in vogue, but the time is past when any man, no matter what his lack of early educational advantages may be, can expect to attain to the highest there is in dentistry. We now think it is better to have our men thoroughly prepared to receive a professional education at the start.

Dr. W. St. George Elliott was pleased to hear the remarks of Dr. Abbott. It had been his privilege to practise dentistry in nearly all parts of the world; he had been a member of English dental societies for eleven years, and he had been sorry to come to the conclusion that the English, in dental education *per se*, were ahead of us. We are their equals in the technique of dentistry, but not in the breadth of scientific education, which with them is the foundation of the dental student's training.

*Dr. W. H. Morgan.*—The success of the practice of dental surgery depends on tactical skill, and the last speaker seems to minify its importance. You can give a man the full science of medicine, and not raise a suspicion of his being skilled in the practice of dentistry.

Dr. Elliott had favored the four years' course as affording greater opportunities for scientific studies, but he did not in any way underrate the necessity for manual skill.

*Dr. Abbott.*—The better prepared the student is to receive his scientific education, the better professional man he will be.

*Dr. E. A. Bogue.*—In view of the remarks of the last three speakers, perhaps it may be well to call attention to the fact that in Austria the student of dentistry must spend seven years in the study of medicine before he can begin the practice of dentistry; hence, there are no good dentists there.

*Dr. Abbott.*—In that country no man can study medicine until after he has graduated from a literary institution, nor dentistry until after he has received his medical education; so that we would naturally say there are no good dentists there. Time does not count there as here. If a man comes through his studies at the age of forty he thinks he is doing well. Here he comes to the college at eighteen and wants to go to work as soon as he can, and at twenty-one or twenty-three he is ready.

Dr. Crouse thought there was one point which college men would do well to consider, and that was as regards undergraduates practising. It is a fact that one-third of those who study dentistry do not practise after getting their education. Anything that would enable these men to find out their unfitness to practise dentistry before they have spent so many years in its study ought to be encouraged. There should certainly be some way of sifting out the unfit material so that a man who was not suited to become a dentist should not use more than a year in finding it out. Perhaps it might do to let them try practice a little before they graduated.

*Dr. J. Taft.*—Perhaps it would not injure the undergraduate but how about his patients? The difficulty is that before a man has completed his studies he is almost necessarily deficient, especially if he is in a school where they have a graded course. The man ought to have a thorough equipment before he is given a chance to treat disease. It is altogether wrong to encourage this idea of undergraduates practising or attempting to practise. Some would go out and win success, and then they would not go back to the college to complete their studies, but would go on with their practice. The best course is to give them the best possible equipment before allowing them to go out to practise. To permit them to begin practice before they are qualified not only results in their personal failure, but the influence of that practice is prejudicial to the good name of the profession.

Dr. W. H. Morgan was willing to accept what had been said in the paper with the explanations that had been given. The broader the education, however, the better qualified is the man to help those in his care ; but, after all, manipulative skill is the key-stone in the arch of dental ability. There was another point in the paper to which he wished to refer. It put forth the idea that a young man who gets into the wrong way, who enters a "cheap John" establishment, should never be admitted to a dental college. Men make mistakes, and it is easy for a young man to make a mistake and get into an establishment of this sort. When he finds it out and wants to rectify it, we ought to take him by the hand and help him ; let him have a chance to recover himself. Again, they say a man ought to be thoroughly qualified before he begins practice. If they mean by this that he should know all he will ever learn about dentistry, he did not believe it possible. The schools ought to be prepared to teach their students sufficient to begin practice intelligently. More than this cannot be expected.

*Dr. Crouse.*—If a student, after one course, takes a position as assistant, does it do him more harm, if he is found incompetent, to tell him so then, than to wait until he has spent another term in a room where there are hundreds of others receiving the same instruction ?

*Dr. James Truman.*—There is a practical side to this question under discussion. Where shall the line be drawn as regards preliminary education ? He did not wish to be put into the ranks of those who are opposed to a high standard of requirements ; but he had become convinced from observation that just in proportion to the extent of his preliminary education is the student defective in technical skill. In a long experience in teaching he had found it impossible to make good technical dentists of students after they reached a certain age. He could, therefore, understand, why it was that in the old countries, where they require the certificate of the gymnasium or the *real schule*, to obtain which takes until the student is twenty to twenty-two years of age, before he can begin his professional studies, and then from five to eight years more must be spent in study,—he could understand why, under these conditions, the manipulative skill of the dentists should not be so high as here. One result of this course has been that in England the dental students, while up in theory, are deficient in practical matters. Now, the question is, Where shall the line be drawn? and he held that until we are prepared to give sufficient qualifications in the common schools to enable the student to comprehend the teaching of the professional studies, the common-school education is where the line should be drawn. We don't want the European standards. What we are after is the making of skilful practical dentists.

*Dr. Abbott.*—The matter of manipulative ability is of the highest importance. Dr. Morgan knew what he was talking about when he suggested that we ought not to keep out the young man who had made the mistake of entering a shyster office. Such a young man frequently, under the right auspices, becomes a shining light because of the manipulative skill he has acquired. What is any professional man good for if he is not a practical man? It is the same with us. The educated dentist without manipulative skill simply wastes his talents because he cannot apply his knowledge. Dr. Truman says that the reason why dentists in Europe are less skilful practically than here is that there they are twenty-four or twenty-five years old before they begin practical work. There is this other reason, that in those countries they lack the genius of manipulation which we have here.

*Dr. E. A. Bogue.*—The genius of manipulation is here because we are a republic, where one man is as good as another, and where practically every one learns to labor with his hands. He himself had the good fortune to be a student in the office of the late Dr. Westcott, and Dr. Westcott's students were taught, first of all, to be mechanics. For two years before they began their studies in college they were taught to use their fingers. He could never forget the things learned there, and he was thankful in his work to-day for the instruction given him then. The school which makes the greatest effort to teach manipulative skill is the one which the experienced dentist selects for the son he intends to train as a dentist.

*Dr. Taft.*—Professor Truman has said that we should draw a line for the study necessary before beginning the dental course. Is that all that is necessary? It would be reasonable to expect that a man claiming to belong to a profession should occupy a respectable position as regards his education. Ten years ago more than one-half the practitioners in dentistry were very defective in their ability to use their native language. It was revealed in their correspondence that there were many who could not write a page which was respectable in its composition. They were not orthographers; they did not possess the ability to construct sentences properly. It is time that this state of things be remedied, and to ask that a reasonable degree of general scholarship shall be possessed by every man who enters into the practice of dentistry. All that is required can be got before the young man is sixteen years of age, and it is not necessary to begin technical study before that time. What is needed is within the reach of every young man in this country, and it ought to be required of them. It is, in fact, a matter which ought to regulate itself. It is not necessary to have what is known by the term thorough culture, although those who have it when they enter college are head and shoulders above those who haven't it. With reference to a knowledge of Latin, which is now required at the entrance examination in some of the colleges, and has been suggested as a requirement in all, it is to be said that we want at least a good knowledge of our own language, and there is no better way to get it than by acquiring a knowledge of those tongues from which it is derived. Up to sixteen or seventeen the student should acquire his general education, which should not interfere with the time which should be devoted to acquiring technical instruction. If the course of training requires the student to go on until he is thirty years old, it is not his previous education, but the crystallization of mind and body which prevents his acquiring manipulative dexterity. Differences in manipulative ability do not depend on degrees of general education.

Dr. John S. Marshall agreed in the main with Dr. Taft's views, but he would go a step further. It stirred his blood to hear members of college faculties get up here and decry the need of a liberal education for dentists. If we are to have a profession of dentists they must have a liberal education, but they must also have medical knowledge. The men who write our text-books are those who have graduated in medicine and then studied dentistry. It is a step backward to oppose a liberal education. Dr. Taft also referred to the lack of general education among dentists. The editor of the *Dental Cosmos* says that what they need is scientific education. If dentists were properly educated these things would not be so. Some one says that we should pay more attention, in the training of dentists, to practical matters than to the theoretical. Dr. Marshall held to the opposite view. The only time for acquiring the theory of dentistry is while the student is at college. He will not spend time upon it after he graduates, but he will get practical skill or he will be a failure as a dentist. It is a mistake to make too much of the practical side. The English dentist is far ahead of us in the theory of dentistry. It would be a good thing if our young men would go there to acquire their theoretical knowledge.

*Dr. Truman.*—Dr. Taft says the mass of dentists are ignorant. That may be true in the West; it is not true in the East. In his position as editor of a dental journal he is brought into contact with large numbers of dentists, and he does not find them ignorant. The papers which come to him as editor would stand very well, compared with those of medical men.

Dr. Taft replied that he did not say that the dentists were a set of ignoramuses. What he did say was that ten years ago there was a great lack of fundamental knowledge among them; but it is not so to-day, and we want out West to get up equal to our brethren in the East.

Dr. Kulp has always advocated a higher order of education as a preliminary to professional studies, more especially for dentistry. It is an open question how to preserve the faculty for acquiring manipulative skill and still have the necessary general culture engrafted. As bearing upon the point, he has found that the young men who have taken a mechanical course in college, which also strengthens and preserves their literary acquirements while giving them a practical knowledge of the use of tools, are the most teachable, when they come to take up the study of dentistry, of any he has ever had.

Dr. T. T. Moore thinks that the solution of the question of a higher education for dentists will be found in the endowment of the colleges. At least one-third of the men now received as students are not competent, and that is the fault of the colleges, or of the system on which they are conducted. The colleges must live upon the fees, and to get these they must have students, and so they keep these men who are not competent; and so long as these conditions prevail institutions will spring up all over the land. The practical solution of the problem is to endow the colleges. When this is done, and the colleges do not have to depend on fees for the payment of running expenses, the intermediate examinations will show the incompetent men,—that is, those who have not the faculties for the successful practice of dentistry,—and they will be dismissed as in military schools. They ought to be allowed the opportunity as early as possible to choose some other calling. It is not right to keep a man three years in a dental college when he is not competent to become a good dentist.

Dr. H. J. McKellops said that he was one of those unfortunates who commenced at the bottom. He was put into a dissecting-room, where he learned the human system, a knowledge which he had always found useful in the practice of dentistry. Now, does the oculist or the aurist ever look into the mouth to see if the teeth are giving trouble? The colleges could do something to raise the standard of dentistry. They could teach thoroughly, for instance, anatomy, not merely of the part in which practical dentistry is most interested, but every part of it; then the students would know anatomy scientifically. Give him the man who can teach science; but if you give the students a lot of patchwork, what can you expect of them in a scientific way? Because students are not thoroughly taught is the reason why there are so many poor dentists. He could hire any number of dentists he chose, graduates of dental colleges, at ten dollars a week. In order to get the best results in teaching students, we have got to start right. Give the student a chance to learn the finer manipulations; don't give him plastic work and expect him to be an ornament to his profession, but cultivate him so that he can do the best things.

Dr. B. H. Catchings has found that the negroes from the dental school in Nashville are thoroughly up in the literary department of their education. They have a written examination, and their answers to questions are nicely expressed. They are, however, as yet deficient in technique.

One feature which shows the general advancement of educational acquirements among dentists is their social position. It is not so many years ago when their social status was about the same as that of the chiropodist of to-day. That was because of the common lack of literary culture. Now they are beginning to be sought after in positions and relations which require the possession of literary ability.

